# Extensively drug-resistant (XDR) *Pseudomonas aeruginosa* identified in Lima, Peru co-expressing a VIM-2 metallo-β-lactamase, OXA-1 β-lactamase and GES-1 extended-spectrum β-lactamase

**DOI:** 10.1099/jmmcr.0.005154

**Published:** 2018-06-21

**Authors:** Paul Ríos, Claudio Rocha, William Castro, Maria Vidal, Enrique Canal, Manuela Bernal, Nathanael D. Reynolds, Drake H. Tilley, Mark P. Simons

**Affiliations:** ^1^​ U.S. Naval Medical Research Unit No.6 (NAMRU-6), Lima, Perú; ^2^​ Centro Médico Naval “Cirujano Mayor Santiago Távara (CEMENA)”, Lima, Perú; ^3^​ Naval Medical Center, San Diego, USA

**Keywords:** carbapenemase, metallo-β-lactamase

## Abstract

**Introduction:**

*Pseudomonas aeruginosa* has the ability to acquire plasmids and other mobile genetic elements that confer resistance to antibiotics. Bacterial genes encoding different β-lactamases (bla), such as metallo-β-lactamases (MBLs) and extended-spectrum β-lactamases (ESBL), can confer resistance to multiple classes of β-lactam antibiotics.

**Case presentation:**

An 83 year old female was admitted in 2012 to the Peruvian Naval Hospital, Centro Médico Naval ‘Cirujano Mayor Santiago Távara’ (CEMENA), in Lima, Peru. A midstream urine sample was collected and sent to the local CEMENA laboratory for routine urine culture. *P. aeruginosa* was isolated and initial antibiotic susceptibility testing showed it to be sensitive to imipenem. The clinicians started a course of meropenem, but the patient did not improve. After 5 days, a second urine culture was performed and a *P. aeruginosa* was isolated again, but this time the strain showed resistance to imipenem. The treatment course was changed to fosfomycin and the patient improved. Phenotypic and molecular laboratory testing to characterize the antibiotic resistance were performed, demonstrating the presence of both MBL and ESBL genes.

**Conclusion:**

To our knowledge, this is the first report of a *P. aeruginosa* XDR clinical isolate that co-expresses an MBL (VIM-2), OXA-1 beta-lactamase and the ESBL (GES-1) in Peru. It is also the first report of a VIM carbapenemase in Peru.

## Introduction

*Pseudomonas aeruginosa* has been well recognized as a cause of nosocomial infection worldwide, principally in urinary tract infections, pneumonias and intra-abdominal infections [1]. Multi-drug resistant (MDR), extensively drug-resistant (XDR), and pan-drug resistant (PDR) bacterial infections have been associated with increased length of hospital stay, multiple morbidities, increased cost of hospitalization and high mortality rates ranging from 50 to 60 % [1–3]. Opportunistic species of the genus *Pseudomonas* have the ability to acquire foreign genetic materials, such as plasmids and other mobile genetic elements. Bacterial genes encoding different β-lactamases (*bla*), such as metallo-β-lactamases (MBLs) and extended-spectrum β-lactamases (ESBL), can confer resistance to multiple classes of β-lactam antibiotics.

MBLs are classified as Ambler class B beta-lactamases based on enzymatic activity that requires a metal ion co-factor to hydrolyze β-lactam antibiotics, including carbapenems. There are more than nine types of MBL, such as IMP, VIM, and NDM that have been reported worldwide including in Africa, Asia, Europe, and the Americas; while others like SPM and GIM have only circulated in specific countries such as Brazil and Germany [4]. We report here the first case of an extensively drug-resistant (XDR) *Pseudomonas aeruginosa* from an 83 year-old female in Lima, Peru that simultaneously expresses the VIM-2, GES-1 and OXA-1 beta-lactamases.

## Case report

An 83 years old female was admitted in 2012 to the Peruvian Naval Hospital, Centro Médico Naval ‘Cirujano Mayor Santiago Távara’ (CEMENA), in Lima, Peru, with complaints of low back pain, vomiting and intestinal obstruction. The patient had a history of high blood pressure, type 2 diabetes mellitus, chronic renal disease and multiple urinary tract infections. In addition, she had a prolapsed bladder with a prior history of hysterectomy. At the time of admission, the patient was taking loperamide, amlodipine and ranitidine.

As part of her initial work-up, a midstream urine sample was collected and sent to the local CEMENA laboratory for routine urine culture. *P. aeruginosa* was isolated and initial antibiotic susceptibility testing showed it to be sensitive to imipenem. The clinicians started a course of meropenem at 500 mg IV q12h based on her renal function, but the patient did not improve. After 5 days, a second urine culture was performed, isolating *P. aeruginosa* that was now resistant to imipenem. She was then changed to renaldosed fosfomycin at 0.8 g IV q12h with noted improvement and clearance of her infection.

The second *P. aeruginosa* isolate was sent to the Naval Medical Research Unit No. 6 (NAMRU-6) in Callao, Peru (coded as MIS1668) for confirmation and further molecular characterization. The isolate was confirmed as *P. aeruginosa* by routine biochemical algorithms and antimicrobial susceptibility testing (AST) was performed using the automated Phoenix System (BD Diagnostics). MIC results were interpreted using the Clinical Laboratory Standards Institute guidelines (CLSI M100-S23) [5]. The isolate was found to be resistant to all antibiotics on the NMIC/ID-124 Phoenix panel (Table 1). Additional antibiotic susceptibilities were tested using the disk diffusion test (DDT) for colistin 10 µg, imipenem 10 µg, and ticarcillin–clavulanic acid 75/10 µg, with only sensitivity to colistin exhibited, thus meeting the criteria for an extensively drug-resistant (XDR) isolate according to the international guidelines [6].

**Table 1.** Antimicrobial susceptibility results

Antibiotic	MIC (µg ml^−1^)*	Interpretation†
Cefepime (FEP)	>16	R
Ceftazidime (CAZ)	>16	R
Meropenem (MEM)	>8	R
Aztreonam (ATM)	>16	R
Piperacillin–Tazobactam (TZP)	>64/4	R
Tobramycin (NN)	>8	R
Amikacin (AN)	>32	R
Gentamicin (GM)	>8	R
Levofloxacin (LVX)	>4	R
Ciprofloxacin (CIP)	>2	R
Imipenem (IPM)	6	R
Ticarcillin–Clavulanic acid (TIM)	6	R
Colistin (CL)	15	S

*MIC: Minimum inhibitory concentration. Results for IPM, TIM and CL correspond to DDT (mm).

†Results obtained according CLSI2013. S, Susceptible; R, Resistant.

**Fig. 1.**
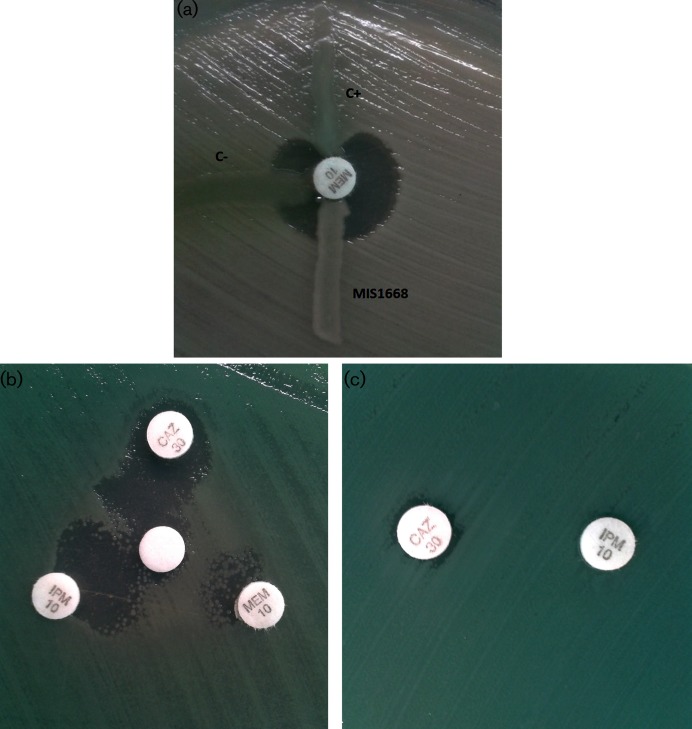
(a). Modified Hodge test for *Pseudomonas aeruginosa* MIS1668. C− (negative control), *Pseudomonas aeruginosa* ATCC 27853. C+ (positive control), *Pseudomonas aeruginosa bla*_IMP-16_. (b). Double disk diffusion test using CAZ (ceftazidime disk), IPM (imipenem disk), MEM (meropenem disk) and EDTA 1M (white disk). (c). Double disk diffusion between CAZ (ceftazidime disk) and IPM (imipenem disk).

Since the MIS1668 isolate was carbapenem-resistant, we used the modified Hodge test (MHT) assay to screen for carbapenemase activity with *Klebsiella pneumoniae* ATCC 700603 [7]. A *P. aeruginosa* harboring the *bla*_IMP-16_ gene and *P. aeruginosa* ATCC 27853 were also used as positive and negative controls, respectively. Surprisingly, MIS1668 was determined to be negative for carbapenemase activity by MHT (Fig. 1a). Thus, we subsequently characterized the isolate using the disk diffusion synergy test (DDST) using EDTA disks with imipenem, meropenem and ceftazidime disks placed 15 mm center-to-center of each disk using the method described by Radice *et al.* [8]. Although MHT was negative, DDST showed synergy when subjecting the isolate to all disks (Fig. 1b), indicating the presence of an MBL enzyme being inhibited by chelation of the metal ion required for activity by the added EDTA. Synergy between imipenem and ceftazidime was observed only in the presence of the EDTA disk, a pattern similar to activity exhibited by the GES enzyme (Fig. 1c) [9].

**Fig. 2.**
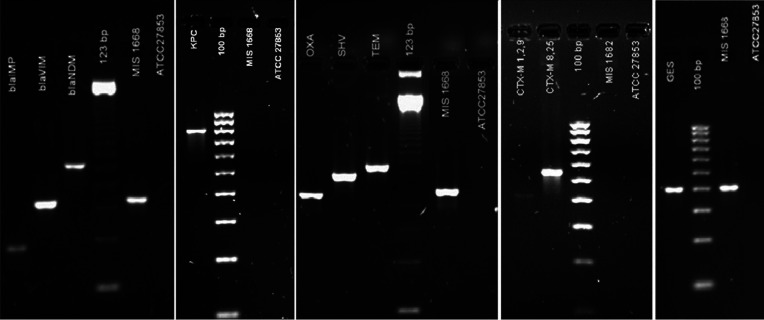
Agarose gel electrophoresis corresponding to PCR for β-lactamase genes. IMP: *bla*_IMP_ positive control. VIM: *bla*_VIM_ positive control. NDM: *bla*_NDM_ positive control. KPC: *bla*_KPC_ positive control. OXA: *bla*_OXA-1-like_ positive control. SHV: *bla*_SHV_ positive control. TEM: *bla*_TEM_ positive control. CTX-M1,2,9 and CTX-M8,25: *bla*_CTX-M_ positive controls. GES: *bla*_GES_ positive control. ATCC 27853: *Pseudomonas aeruginosa* strain used as a negative control. 123 bp: Ladder of 123 base pars. 100 bp: Ladder of 100 base pars.

To further characterize the carbapenem-resistance activity, we performed a combination of multiplex PCR to detect *bla* genes with primer sets for the MBLs (IMP, VIM, NDM), serine-carbapenemase KPC, and for other beta-lactamases (TEM, SHV, OXA, GES, VEB, PER and CTX-M) using the primers listed in Table 2. The series of multiplex PCRs clearly demonstrated positive bands for *bla*_VIM_, *bla*_GES_ and *bla*_OXA-1-like_ from the *P. aeruginosa* MIS1668 isolate with band sizes consistent with those of the controls (Fig. 2). Subsequently, we used Sanger-based sequencing (Applied Biosystems) of the amplicons followed by blast search of the sequences to confirm the *bla*_VIM-2_, *bla*_GES-1_ and *bla*_OXA-1_ gene variants (Table S1 available in the online version of this paper). Finally, we used the Diversilab Rep-PCR method (Biomerieux) to detect homogenous DNA regions in the bacterial genome to the reference strain ATCC 27853 and found the MIS1668 isolate to be 94.1 % homologous.

**Table 2.** Primers used to detect *bla* genes

Gene	Primer	Sequence (5′–3′)
*bla*_IMP_	IMP-F	GGAATAGAGTGGCTTAAYTCTC
	IMP-R	GGTTTAAYAAAACAACCACC
*bla*_VIM_	VIM-F	GATGGTGTTTGGTCGCATA
	VIM-R	CGAATGCGCAGCACCAG
*bla*_NDM-1_	NDM-F	GGTTTGGCGATCTGGTTTTC
	NDM-R	CGGAATGGCTCATCACGATC
*bla*_KPC_	KPC-F	CGTCTAGTTCTGCTGTCTTG
	KPC-R	CTTGTCATCCTTGTTAGGCG
*bla*_TEM_	TEM-F	CATTTCCGTGTCGCCCTTATTC
	TEM-R	CGTTCATCCATAGTTGCCTGAC
*bla*_SHV_	SHV-F	AGCCGCTTGAGCAAATTAAAC
	SHV-R	ATCCCGCAGATAAATCACCAC
*bla*_OXA-1-like_	OXA-1F	GGCACCAGATTCAACTTTCAAG
	OXA-1R	GACCCCAAGTTTCCTGTAAGTG
*bla*_GES_	GES-F	AGTCGGCTAGACCGGAAAG
	GES-R	TTTGTCCGTGCTCAGGAT
*bla*_PER_	PER-F	GCTCCGATAATGAAAGCGT
	PER-R	TTCGGCTTGACTCGGCTGA
*bla*_VEB_	VEB-F	CATTTCCCGATGCAAAGCGT
	VEB-R	CGAAGTTTCTTTGGACTCTG
*bla*_CTX-M-1,2,9_	CTX-MA1	SCSATGTGCAGYACCAGTAA
	CTX-MA2	CCGCRATATGRTTGGTGGTG
*bla*_CTX-M-8,25_	CTX825F	CGCTTTGCCATGTGCAGCACC
	CTX825R	GCTCAGTACGATCGAGCC

## Discussion

Bacterial isolates simultaneously carrying multiple β-lactamases of different molecular classes, such as the ESBL (molecular class A or D) and MBL (molecular class B), are rare events. Such reports of *P. aeruginosa* isolates with genes coexisting and simultaneously encoding MBLs and ESBLs include: VIM-2 with CTX-M-2 in Uruguay, VIM-2 with OXA-10 in Croatia and IMP-1 with CTX-M-2 and IMP-1 with GES-1 in Brazil [10–12]. Also there is one report of the coexistence of VIM-2 and GES-1 in Argentina [13] within a *P. aeruginosa* strain very similar to the strain isolated in this case, indicating that these resistance factors could have originated in South American countries.

Although treatment efficacy for MBL *in vivo* remains unknown and carbapenems could still have some activity, in this case meropenem treatment clinically failed [14, 15]. It is also possible that this *P. aeruginosa* isolate has resistance mechanisms other than beta-lactamases, such as efflux pumps, that may be contributing to its carbapenem resistance. However, it has been shown in hospitals in Belgium that all *P. aeruginosa* isolates expressing VIM-2 showed high levels of cross resistance to both imipenem and meropenem as in this case [16]. However, this patient was successfully treated with fosfomycin, which works to disrupt the first step in the biosynthesis of the bacterial cell wall by the formation of the peptidoglycan precursor uridine diphosphate N-acetylmuramic acid [17]. Although there is some experience in using fosfomycin in the treatment of MDR urinary tract infections, there is less so in more invasive or systemic infection [18]. Given the ability of *P. aeruginosa* to easily acquire resistance to fosfomycin without a cost to its overall fitness, it would be cautious to avoid treating systemically ill patients with fosfomycin monotherapy until there is more clinical outcome data available [19, 20].

Although the MIS1668 isolate harbors a *bla*_VIM_ gene encoding a carbapenemase, as detected by molecular tests, the isolate was negative for carbapenemase activity by MHT. In our case, the use of EDTA to chelate the zinc ions to allow inhibition of the enzyme demonstrated the carbapenemase phenotype but showed the importance of additional phenotypic tests in screening for a MBL and the need for molecular confirmatory testing [21].

The results of the Diversilab rep-PCR based comparison revealed that the *P. aeruginosa* presented here showed a 94 % homology with the reference carbapenem-sensitive ATCC 27853 strain, and indicate that this isolate has picked up an integron or possibly a gene cassette over time with a relatively conserved chromosome [22]. Although the initial isolate that was reportedly sensitive to imipenem was not saved for further testing, we presume that the isolate probably acquired genes necessary for carbapenem resistance through the sharing of plasmids at the time of treatment. However, it would be safe to assume that the antimicrobial pressure over time most probably led to the sharing of transposons between bacteria, leading to the current isolate within this particular hospital. This could be further investigated by comparing other carbapenemase-resistant *P. aeruginosa* isolates within Peru to see if these genes are indeed conserved and prevalent.

To our knowledge, this is the first report of a *P. aeruginosa* XDR clinical isolate that co-expresses an MBL (VIM-2), OXA-1, and an ESBL gene (GES-1) in Peru. It is also the first report of a VIM carbapenemase in Peru although this has been described in other countries in South America. Given its presence in other neighboring countries, the full extent of MBL prevalence in Peru is unknown and is a topic of targeted surveillance for our program and others. Most infection control programs at large hospitals in Peru are managed by professionals with strong clinical backgrounds but with limited resources to provide effective infection control monitoring and limited laboratory capability to reliably detect increasing bacterial resistance rates. Developing and strengthening reliable surveillance systems to identify isolates with genes encoding antibiotic resistance is essential in developing effective infection control polices, informing antimicrobial stewardship practices and improving overall clinical care. To further understand how these resistance mechanisms disseminate through mobile genetic elements and to develop novel interventions to counter these undesirable gene flows, robust surveillance systems are needed among laboratories in developing countries. This case illustrates the priority to establish these enhanced surveillance networks and to implement them in more resource-limited areas where multi-drug-resistant isolates may be developing and circulating.

## Supplementary Data

Supplementary File 1
