# Supplementary material for: Extensively drug-resistant (XDR) Pseudomonas aeruginosa identified in Lima, Peru co-expressing a VIM-2 metallo-β-lactamase, OXA-1 β-lactamase and GES-1 extended-spectrum β-lactamase
Source: JMM Case Rep. 2018 Jun 21;5(7):e005154. doi: 10.1099/jmmcr.0.005154 (PMC6159545; doi:10.1099/jmmcr.0.005154)
Supplement: Supplementary File 1 [file jmmcr-5-5154-s001.pdf]

Supplementary Table 1.

Gene	Amplicon Sequence
bla$_{VIM}$	GATGGTGTTTGGTCGCATATCGCAACGCAGTCGTTTGATGGCGCAGTCTACCCGTCCAAT<br>GGTCTCATTGTCCGTGATGGTGATGAGTTGCTTTTGATTGATACAGCGTGGGGTGCGAA<br>AAACACAGCGGCACTTCTCGCGGAGATTGAGAAGCAAATTGGACTTCCTGTAACGCGTG<br>CAGTCTCCACGCACTTTCATGACGACCGCGTCGGCGGCGTTGATGTCCTTCGGGCGGCTG<br>GGGTGGCAACGTACGCATCACCGTCGACACGCCGGCTAGCCGAGGTAGAGGGGAACGA<br>GATTCCCACGCACTCTCTAGAAGGACTCTCATCGAGCGGGGACGCAGTGCGCTTCGGTC<br>CAGTAGAACTCTTCTATCCTGGTGCTGCGCATTCGA
bla$_{GES}$	AGTCGGCTAGACCGGAAAGAGCCGGAGATGGGCGACAACACACCTGGCGACCTCAGAG<br>ATACAACTACGCCTATTGCTATGGCACGTACTGTGGCTAAAGTCCTCTATGGCGGCGCAC<br>TGACGTCCACCTCGACCCACACCATTGAGAGGTGGCTGATCGGAAACCAAACGGGAGAC<br>GCGACACTACGAGCGGGTTTTCCTAAAGATTGGGTTGTTGGAGAGAAAACTGGTACCTG<br>CGCCAACGGGGGCCGGAACGACATTGGTTTTTTTAAAGCCCAGGAGAGAGATTACGCTG<br>TAGCGGTGTATACAACGGCCCCGAAACTATCGGCCGTAGAACGTGACGAATTAGTTGCC<br>TCTGTCGGTCAAGTTATTACACAACTCATCCTGAGCACGGACAAAA
bla$_{OXA\text{-}1\text{-}like}$	TGGCACCAGATTCAACTTTCAAGATCGCATTATCACTTATGGCATTTGATGCGGAAATAA<br>TAGATCAGAAAACCATATTCAAATGGGATAAAACCCCCAAAGGAATGGAGATCTGGAAC<br>AGCAATCATACACCAAAGACGTGGATGCAATTTTCTGTTGTTTGGGTTTCGCAAGAAATA<br>ACCCAAAAAATTGGATTAAATAAAATCAAGAATTATCTCAAAGATTTTGATTATGGAAAT<br>CAAGACTTCTCTGGAGATAAAGAAAGAAACAACGGATTAACAGAAGCATGGCTCGAAA<br>GTAGCTTAAAAATTTCACCAGAAGAACAAATTCAATTCCTGCGTAAAATTATTAATCACA<br>ATCTCCCAGTTAAAAACTCAGCCATAGAAAACACCATAGAGAACATGTATCTACAAGATC<br>TGGAGAATAGTACAAAACTGTATGGGAAAACTGGTGCAGGATTCACAGCAAATAGAACC<br>TTACAAAACGGATGGTTTGAAGGGTTTATTATAAGCAAATCAGGTCATAAATATGTTTTT<br>GTGTCCGCACTTACAGGAAACTTGGGGTCA
